# A History of the Discovery of Random X Chromosome Inactivation in the Human Female and its Significance

**DOI:** 10.5041/RMMJ.10058

**Published:** 2011-07-31

**Authors:** Sophia Balderman, Marshall A. Lichtman

**Affiliations:** 1 Department of Medicine, University of Rochester Medical Center, Rochester, New York, USA and; 2 Departments of Biochemistry and Biophysics, University of Rochester Medical Center, Rochester, New York, USA

**Keywords:** X chromosome, gene dosage, glucose-6-phosphate dehydrogenase, clonality

## Abstract

Genetic determinants of sex in placental mammals developed by the evolution of primordial autosomes into the male and female sex chromosomes. The Y chromosome determines maleness by the action of the gene *SRY*, which encodes a protein that initiates a sequence of events prompting the embryonic gonads to develop into testes. The X chromosome in the absence of a Y chromosome results in a female by permitting the conversion of the embryonic gonads into ovaries. We trace the historical progress that resulted in the discovery that one X chromosome in the female is randomly inactivated in early embryogenesis, accomplishing approximate equivalency of X chromosome gene dosage in both sexes. This event results in half of the somatic cells in a tissue containing proteins encoded by the genes of the maternal X chromosome and half having proteins encoded by the genes of the paternal X chromosome, on average, accounting for the phenotype of a female heterozygote with an X chromosome mutation. The hypothesis of X chromosome inactivation as a random event early in embryogenesis was first described as a result of studies of variegated coat color in female mice. Similar results were found in women using the X chromosome-linked gene, glucose-6-phosphate dehydrogenase, studied in red cells. The random inactivation of the X chromosome-bearing genes for isoenzyme types A and B of glucose-6-phosphate dehydrogenase was used to establish the clonal origin of neoplasms in informative women with leiomyomas. Behind these discoveries are the stories of the men and women scientists whose research enlightened these aspects of X chromosome function and their implication for medicine.

## INTRODUCTION

This historical review describes the men and women, their ideas, their discoveries, and their interactions, which ultimately resulted in the recognition and understanding of the random inactivation of the paternal- or maternal-derived X chromosome in the somatic cells of women. This year, 2011, is the 50th anniversary of the exposition of the hypothesis of random inactivation of the X chromosome in mammalian females. We also review the use of an X chromosome-linked gene product, glucose-6-phosphate dehydrogenase (G6PD), to further our understanding of gene dosage regulation, heterogeneity in the expression of X chromosome-linked traits, and as a tool to establish the single cell derivation of a neoplasm. The last-mentioned approach provided an understanding of how the qualitative tissue alteration, neoplasia, differs from all other principal tissue abnormalities: hypoplasia, hyperplasia, dysplasia, and metaplasia.

## EVOLUTION OF SEX CHROMOSOMES IN EUTHERIAN (PLACENTAL) MAMMALS

At some point in the evolution of more complex organisms, gametes developed containing the haploid complement of chromosomes. One was usually a motile gamete capable of searching out and penetrating the immobile gamete (fertilization), the latter containing nutrients capable of sustaining the early phases of individual development.^1^ In the most rudimentary forms of bisexuality, maleness or femaleness had a random quality; but, in mammals, bisexuality was dependent on the development of two distinct chromosomes, one committed to maleness and one permitting the development of femaleness. These unique chromosomes were derived from a homologous pair of autosomes over evolutionary time and became specialized into sex chromosomes approximately 100 million years ago.^1^

In the specific case of mammals, the X chromosome has shed most of the genes allelic to the genes on its antecedent primordial autosome. The Y chromosome is a profoundly atrophied version of the primordial autosome from which it derived, having lost most of its genes (over one thousand) since it was no longer capable of recombining over most of its length with a paired chromosome during meiosis in males. Thus, the principal way to eliminate deleterious genes on the Y chromosome was to delete them, leading over millions of years to a shrunken chromosome.^1^–^3^ The Y chromosome’s key gene is the sex-determining region Y gene or *SRY*. It encodes the testes-determining factor (TDF) or SRY protein, a transcription factor that initiates a cascade of biological events resulting in the conversion of the embryonic gonads into testes and, thereby, male sex determination. Thus, the sex of a human is determined by the development of testes under the influence of a Y chromosome and development of ovaries in the presence of an X chromosome in the absence of the Y. In disorders of chromosome non-disjunction an XXY individual is a phenotypic male and an XO individual is a phenotypic female. Rarely, inactivating mutations in the *SRY* gene give rise to XY females with gonadal dysgenesis; and translocation of the segment of the Y chromosome carrying *SRY* to the X chromosome causes the XX male syndrome.

## IDENTIFYING THE X CHROMOSOME

The X and Y chromosome are the only two that have a letter designation. All the human autosomes are designated by a number. Naming the X chromosome is attributed to Karl Heinrich Henking (1855–1934), a German biologist, who while studying wasp sperm cell mitosis, noted that some sperm cells had 12 chromosomes whereas others had 11. He also observed that during meiotic divisions the 12th chromosome had a somewhat different morphologic appearance. He designated the 12th chromosome the “X element”, presumably using the mathematician’s symbol for an unknown quantity. He speculated on its possible role in sex determination in insects, but it was the American biologist Charles Erwin McClung (1870–1946) who studied what he referred to as the “accessory chromosome” in the process of spermatogenesis in various organisms. Working in the laboratory of zoology and histology at the University of Kansas in Lawrence at the turn of the twentieth century, he was particularly struck by the behavior of the accessory chromosome during the first meiotic division in grasshopper spermatogenesis. Here four sperm cells result from one spermatogonium, but only two of four sperm cells contain the accessory chromosome. He found this pattern of segregation during spermatogenesis in other organisms and reasoned that the segregation of the accessory chromosome to half the sperm cells meant that this effect would result in two types of zygotes, one with and one without the accessory chromosome. Since only one characteristic, gender, was usually allocated in a 50:50 distribution in zygotes, he concluded that the accessory chromosome was a sex determinant.^4^ He also concluded, erroneously, that the accessory chromosome determined male sex and the lack of the accessory chromosome resulted in a female offspring.

Walter Stanborough Sutton (1877–1916) did meticulous studies of the chromosomes of grasshoppers, counting and sizing each chromosome, arraying them by size, and measuring them throughout meiosis. He found that sperm cells contain half the number of chromosomes of the grasshopper zygote. He provided evidence that the chromosomes in the zygote were the result of the coming together of the chromosomes in each gamete, resulting in chromosome pairs, and that the X element or accessory chromosome was a legitimate chromosome, despite its disparate segregation in gametes. The appreciation that certain chromosomes, based on their 50:50 distributions in gametes, may be the determinants of sex was the initial evidence that chromosomes carried heritable traits. His paper on the role of chromosomes in heredity, although based in part on the work of some predecessors, including Mendel, was the most carefully reasoned to that time, and he is credited with the articulation of the chromosome theory of heredity.^5^ Sutton received his bachelor’s and master’s degree at the University of Kansas. His master’s thesis on spermatogenesis in the grasshopper was conducted under the guidance of his mentor McClung. He started but did not complete his PhD degree at Columbia University, but after two subsequent years in Kansas developing devices related to oil-drilling (he had earlier training in engineering and mechanical skills), he returned to New York City and received a degree in medicine from the Columbia University College of Physicians and Surgeons. He practiced surgery for the rest of his short life, making several notable contributions to the field.

At the turn of the twentieth century, scientists began reporting a mismatched pair of chromosomes in the cells of male beetles. Edmund Beecher (E.B.)Wilson (1856–1939), a pre-eminent zoologist at Columbia University in New York City, found equal numbers of chromosomes in male and female cells in several species of insects, but one pair of chromosomes in male cells was disparate in size. He believed the larger of the mismatched pair was the X element of Henking and accessory chromosome of McClung and that the smaller chromosome was unapparent to his predecessors because of its size.

Nettie Maria Stevens (1861–1912) at Bryn Mawr College, studying the mealworm, uncovered the fact that female cells had 20 large chromosomes and male cells had 19 comparably large chromosomes but always in addition a small one. She also found that female eggs had 10 large chromosomes but male sperm had either 10 large or 9 large and 1 small chromosomes. She realized that the small chromosome was the partner of the accessory chromosome (future X chromosome) and it was probably the determinant of sex, based on its absence or presence. Stevens spent most of her life trying to become a scientist. She worked as a teacher and librarian for years to save sufficient money to do so. At age 35, she entered Leland Stanford University in California. Thereafter, she began graduate school at Bryn Mawr College and received her doctorate degree at age 42. She remained there to pursue her interest in biological research, spending periods at Cold Springs Harbor Laboratories as well. She died from breast cancer 9 years after receiving her advanced degree but made seminal contributions to the understanding of sex determination and chromosome function by dint of her indefatigable character and commitment to science in her short, but remarkable, career.

## EARLY GENETIC DISCOVERIES THROUGH BOTANICAL RESEARCH

Genetics began to flourish at the turn of the twentieth century. The implications of the earlier profound discoveries by Gregor Johann Mendel (1822–1884), an Augustine monk and technical high school teacher, in the mid-nineteenth century were ignored by the scientific community for over three decades.^6^ One explanation given for Mendel’s obscurity was the effect of the publication of Charles Robert Darwin’s (1809–1882) *On the Origin of Species* in 1859 and the dominance that the topic of evolution and the process of natural selection had in scientific discourse over the ensuing decades. This explanation has been vigorously disputed, and alternative explanations include the minimal circulation of the publication describing Mendel’s findings and conclusions, his failure to continue his research after he was elevated to abbot 2 years later, and the failure of his colleagues in botany to recognize the significance of his work. Nevertheless, the ascension of genetics in the first years of the twentieth century was propelled by the rediscovery of Mendel’s findings, virtually simultaneously, by two scientists in 1900: Hugo Marie De Vries (1848–1935) in Holland and Carl Correns (1864–1933) in Germany.^7^ They each published results that coincided with Mendel’s deductions and in the process of searching the literature found Mendel’s report to which they gave scientific priority. They confirmed Mendel’s insights, based on his experiments with the garden pea plant, that units of information in parents account for observable traits in offspring and are passed from one generation to the next. Mendel promulgated the idea that inherited factors do not combine (blend), but are passed intact; each member of the parental generation transmitting only half of its hereditary factors to each offspring and, thus, different offspring of the same parents may receive different combinations of hereditary factors in predictable ratios. Certain factors were dominant, explaining their more frequent expression in the offspring. His two lectures on plant hybridization to the Natural History Society of Brünn, Moravia in the Austro-Hungarian Empire (now Brno in the Czech Republic) in February and March 1865 and the ensuing monograph *Versuche über Pflanzen-Hybriden* (*Experiments in Plant Hybridization*) published in 1866 in the Transactions of that Society described his most important findings. These ideas lay fallow for over three decades. Mendel predicted that his work would eventually be found to be of importance, although he did not live to see its rediscovery. His treatise, rediscovered, was reprinted and distributed widely and translated into English by the Royal Horticultural Society in 1901. Mendel, also, considered hybridization capable of contributing to speciation and supported Darwin’s theory of evolution but provided an additional mechanism for its conduct.^8^

Wilhelm Ludvig Johannsen (1857–1927), a Danish chemist and botanist, confirmed the work of the Dutch plant biologist de Vries that rapid changes in discrete particles of heredity (mutations) in the germ cell resulted in phenotypic changes. Johannsen abbreviated de Vries’s term “pangene” to “gene” (*gen* in Danish, anglicized to *gene*) as a descriptor of these discrete particles that represented the units of information described by Mendel. He also distinguished the genotype from the phenotype of an individual.^9^ Mendel recognized that the heritable factors that controlled a specific trait could take more than one form (e.g. maternal and paternal), and Johannsen named the different forms of a gene *allelomorphs*, later shortened to *allele*. Johannsen revised and lengthened an earlier treatise after he became aware of Mendel’s studies and published *Arvelighedslaerens elementer* (The Elements of Heredity) in 1905. An expanded German edition became available in 1909 and became the most influential text on genetics in Europe. The book’s emphasis on quantification and statistical methods made it one of the most authoritative texts of the day.

## THE EARLY APPLICATION OF THE LAWS OF INHERITANCE TO HUMAN DISEASE

The application of inheritance to medicine was highlighted at this time by Archibald Edward Garrod (1857–1936), who recognized the genetic basis of certain diseases of metabolism and connected an abnormality in inheritance to specific biochemical disorders that he attributed to defective enzymes.^10^ In 1908, the core of his work was presented at the Croonian Lectures entitled “Inborn Errors of Metabolism” to the Royal College of Physicians in London and was published the following year. Garrod’s metabolic studies included consideration of alkaptonuria, cystinuria, pentosuria, and albinism. He succeeded William Osler (1849–1919) as Regius Professor of Medicine at Oxford in 1921. In 1923, he summarized his biochemical and phenotypic studies in an expanded edition of his classic text, *Inborn Errors of Metabolism*. He ascribed most enzyme defects to Mendelian recessive inheritance.

## FINDING SEX LINKAGE OF PHENOTYPIC TRAITS

In 1910, Thomas Hunt Morgan (1866–1945), the American biologist and embryologist, first reported an X chromosome (sex)-linked trait. He was on the faculty of Columbia University from 1904 to 1926 and chose *Drosophila melanogaster*, the fruit fly, as his experimental model for his pioneering studies of inheritance. The ability to study many generations in a short period of time and the ease of maintenance of the species (low cost and simplicity) was appealing to the academic biologist. He attracted outstanding students to his laboratory, dubbed the “Fly Room”, who contributed to several key discoveries. Early during his research, he noticed a male fly with white eyes, an unusual finding as the eyes of *Drosophila* are usually red. He did a series of breeding experiments starting with the white-eyed male and a red-eyed female. Morgan’s suspicion of sex-linkage of eye color in *Drosophila* increased as his experiments fit that hypothesis. At the end of his series of cross-breeding experiments, Morgan concluded that the white-eyed trait followed a sex-linked pattern of inheritance as opposed to a typical Mendelian pattern of inheritance (i.e. dominant or recessive).^11^ Morgan’s laboratory conducted pioneering studies of gene linkage and crossing-over. This work led to the development of genetic maps and the resultant determination of the relative position of genes on a given chromosome.

In 1933, Morgan was the first American scientist to win a Nobel Prize, specifically the Nobel Prize for Physiology or Medicine, for his studies of the genes in *Drosophila* and his several discoveries including the rules of recombination.^12^ Based on recombinant frequency, the unit designated the Morgan or, more frequently, the centiMorgan or map unit, became a measure of genetic linkage. It was named by his student and, later, career-long colleague, Alfred Henry Sturtevant (1891–1970), who constructed the first genetic map of a chromosome in 1913 and, thereby, developed the chromosome mapping technique. Morgan was nominated for the Nobel Prize in 1919 and 1922, but at that time genetic studies were not considered either physiology or medicine, and he was not given primary consideration for the Prize. In 1927, he was recruited to the newly established California Institute of Technology (originally Throop College) in Pasadena, California in order to reorganize the biology department at the request of Robert Andrews Millikan (1868–1953), effectively its first president and a Nobel Prize Laureate in Physics in 1923. It was part of a successful, focused effort to develop one of the great scientific institutions of higher education in the United States. Although converted in part to an administrator, Morgan had brought several of his research protégés with him; among them was Sturtevant who led a genetics group at Cal Tech. In the summer of 1933, while at Cal Tech, Morgan received an honorary doctor of medicine degree from the University of Zurich for his contributions to genetics and to medicine. This event and the profundity of his laboratory’s discoveries awakened the Nobel Prize selection committee in Physiology or Medicine to the relevance and importance of his studies to human genetics and medicine. They responded by announcing his selection for the Prize in October of 1933, the month of Alfred Nobel’s (1833–1896) birth, and awarded it to him on December 10th, 1933, the month and day of Nobel’s death, as is the practice of the Nobel Foundation.^12^

## IDENTIFYING THE HETEROCHROMATIC X CHROMOSOME IN THE NUCLEUS OF ANIMAL AND HUMAN FEMALE CELLS

**Figure 1:**
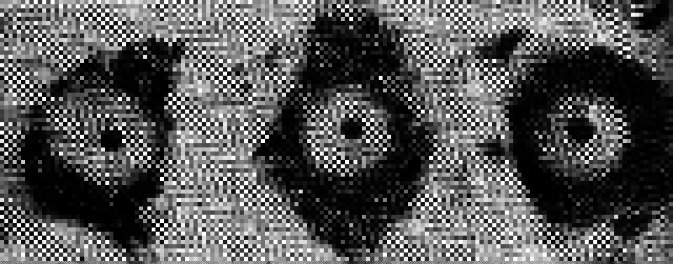
Left: Normal motor neuron from the hypoglossal nucleus of a mature female cat showing the usual morphology of the nucleolar satellite (indicated by arrow) in the female. Cresyl violet stain, × 1,400. Middle: Motor neuron from the hypoglossal nucleus of a mature male cat. The nucleolar satellite is absent, the typical condition in the mature male. Cresyl violet stain, × 1,400.Right: Motor neuron from the hypoglossal nucleus of a mature female cat 108 hours following electrical stimulation of the corresponding hypoglossal nerve for a period of 8 hours. Associated with intense synthesis of cytoplasmic ribose nucleoproteins, the nucleolar satellite (indicated by arrow) tends to move away from the nucleolus. Cresyl violet stain, × 1,400. (From: Barr ML, Nature 1949;163:676, with permission from Nature Publishing Group.^13^)

In 1949, Murray Llewellyn Barr (1908–1995) and his graduate student Ewart George Bertram (b. 1923) of the University of Western Ontario, London, Ontario made an unexpected discovery that would eventually further our understanding of X chromosome function. Barr was interested in the effect of fatigue in airmen on their brain function. Barr and Bertram performed a study sponsored by the Institute of Aviation Medicine of the Royal Canadian Air Force (Barr had been a medical officer in the Royal Canadian Air Force during World War II) to determine whether nerve stimulation led to detectable neuronal cytological changes. The cat neurons were exposed to the Fuelgen reagent, which stains DNA. They observed the presence of a darkly stained aggregate of DNA in some but not other neuronal cells. They eventually realized that the interphase nuclei of the neurons of the female but not the male cat contained the aggregate. They initially called the intensely staining area a “nucleolar satellite” (Figure 1). The nucleolar satellite was so named because of its proximity to the nucleolus in these cells. Barr and Bertram hypothesized that the morphologic difference in the intermitotic nuclei of differentiated cells between males and females was a reflection of the heterochromatin of the sex chromosomes. They also reported that “preliminary examination of sympathetic ganglia … indicates a similar sex difference in nuclear morphology exists in the human”.^13^ This sexual dimorphism of the cell nucleus was later shown by Keith Leon Moore (b. 1925) and Barr to exist in cells of non-nervous tissue and across a wide variety of mammalian species.^14^ In 1959, Barr published a paper in the journal *Science* reviewing the then current knowledge of sexual development and the questions relating to a “special mass of chromatin” or the “sex chromatin”, as he then referred to it.^15^ (Later, it would be given the eponymous (and euphonious) designation the “Barr body” after its discoverer.)Barr was unable to reconcile the phenotypes in Turner’s and Klinefelter’s syndromes with the morphologic appearances of their nuclei since patients with Turner’s syndrome (XO), though lacking a nuclear sex chromatin, have a female phenotype, whereas patients with Klinefelter’s syndrome (XXY) have a sex chromatin in their interphase nuclei but a male phenotype. This conundrum was based on his supposition that the Y chromosome was passive in sex determination. After submission of Barr’s paper to *Science*, other data were published indicating that the Y chromosome was central in determining the male phenotype. In an addendum to the paper, Barr briefly reviewed these new data and, thereby, reconciled the female phenotype of Turner’s syndrome and the male phenotype of Klinefelter’s syndrome with the absence of the sex chromatin in the former and the presence of it in the latter.^15^

Barr initially believed that the sex chromatin represented the heterochromatic portion of the two X chromosomes that were somatically paired, and that the XY were together too small to create such an effect. This explanation was found not to be the case, as will be discussed below, and in a letter to the journal *Science* Barr realized that other data did not support his concept of somatically paired X chromosomes representing the sex chromatin.^16^

**Figure 2:**
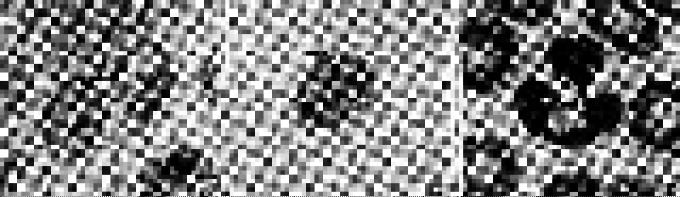
Nuclei of human females. A: Nuclei in epidermis of a skin biopsy specimen (hematoxylin-eosin); B: nucleus in an oral mucosal smear (cresyl echt violet); C: neutrophil in a blood film (Giemsa)(× about 1,680). (From: Barr ML, Brit J Urol 1957;29:251, with permission from John Wiley & Sons.)

Barr and Moore devised a buccal mucosa scraping technique for sex determination. It was a relatively simple diagnostic test for identification of gender, in which cells scraped from the lining of the oral cavity were fixed, stained, and examined microscopically for the condensed area in the nucleus that represented the sex chromatin in an individual with two X chromosomes.^17^ Using this technique, confirmation of the diagnosis of individuals suspected of certain sex chromosome abnormalities could be made (Figure 2). For example, Turner’s syndrome, in which phenotypic females have only one X chromosome (XO), could be confirmed by the lack of a sex chromatin in the patient’s buccal mucosal cells, whereas phenotypic males with Klinefelter’s syndrome had a sex chromatin in their mucosal cells, representing the additional X chromosome (XXY). The test was used by the International Olympic Committee from 1968 to 1991 to verify the gender of athletes. The test’s use led to the dismissal of several male athletes who had falsified their gender. The test was supplanted by the polymerase chain reaction method for the *SRY* gene on the Y chromosome. In 2000, the Olympic Committee stopped testing all athletes for gender and now does so only on a case-by-case basis.

Barr, on the faculty of the medical school at the University of Western Ontario, became professor of microscopic anatomy in 1952 and later chair of the department. He was the recipient of numerous honors and awards for his work. He also authored an authoritative text, *The Human Nervous System: An Anatomical Viewpoint* in 1972, which was updated by Barr for several subsequent editions.^18^,^19^

The further exploration of the significance of the sex chromatin and its relationship to the X chromosome in the female was dependent, in part, on development of chromosome analysis, especially in human cells, and the availability of a product (marker) of an X chromosome-linked gene that would permit a determination of X chromosome-linked gene expression.

## DELINEATING CHROMOSOME NUMBERS AND APPLYING CYTOGENETIC TECHNIQUES

In 1949, the time of Barr’s discovery of the nuclear body in the cells of females, reliable methods for examining human chromosomes had not been developed. Although an interest had been kindled in counting chromosomes at the end of the nineteenth century, techniques were flawed, and the clumping of chromosomes in cell preparations led to gross under-estimations of the human diploid number. Walther Flemming (1843–1905), a German physician and biologist, is credited with the discovery of chromosomes (Greek for colored body), so named because of their staining with aniline dyes, made available by the burgeoning German chemical industry in the late nineteenth century. He named the nuclear structures that took up the basophilic dye “chromatin” (colored material), which he identified as thread-like structures in the nucleus.^20^ In 1888, Heinrich Wilhelm Gottfried von Waldeyer-Hartz (1836–1921)(a.k.a. Heinrich Wilhelm Waldeyer), a German anatomist for whom the lymphatic ring in the oro-nasopharynx and other structures are named, coined the term c*hromosomen* (colored bodies) to describe Flemming’s nuclear threads (chromatin).^21^ In his studies of cell division, Flemming proposed the term *mitosen* (Greek for thread) for the process in which linear, thread-like structures formed in the nucleus and were distributed to daughter cells. Flemming worked at the German University in Prague and then the University of Kiel, where he became director of the Anatomical Institute. In 1882, he published his text, *Zellsubstanz, Kern und Zelltheilung* (*Cell Substance, Nucleus, and Cell Division*), which described much of what we know today about the physical process of mitosis. He deduced that cell nuclei arose from predecessor nuclei, calling the process *omnis nucleus e nucleo* (all nuclei come from nuclei) after Rudolf Carl Virchow’s (1821–1902) phrase *omnis cellula e cellula* (all cells come from cells).

In 1912, Hans de Winiwarter (1875–1949), a gynecologist and professor in Liege, Belgium who had an interest in the nature of gender determination, reported from his studies of fetal ovaries that the human diploid chromosome number was probably 48. In the testes, he counted 47 chromosomes.^22^ Although his counts would later prove imprecise, he had improved methods sufficiently to put to rest the prior reports of human chromosome diploid numbers in the twenties.

In 1921, Theophilus Shickel Painter (1889–1969) an American biologist, cytologist, and geneticist, who had studied with Theodor Heinrich Boveri (1862–1915) in Würzburg as part of a post-doctoral year in Europe, reported his studies of human chromosomes. Painter spent his early years at Yale University in New Haven, Connecticut and moved to the University of Texas in Austin, where he rose to professor and later became its president. He deviated from his usual species of interest, *Drosophila*, for a time to study primate and human tissue. He published an influential paper in the journal *Science* describing the Y chromosome in human testicular cells (he had already done so in the opossum) and concluded that the sex chromosome composition in men was XY. From his observations, he concluded that the number of chromosomes in human testicular cells was between 45 and 48, and probably 46 or 48. He states:“… in the clearest equatorial plates so far studied only 46 chromosomes have been found”.^23^ He obtained human testicular tissue from a nearby psychiatric hospital where orchiectomy was sometimes performed for “therapeutic reasons”.^24^ He could obtain fresh tissue and fix it rapidly through the agency of a former premedical student at the University who was a physician at that hospital.

In the early twentieth century, techniques for examining chromosomes in cells were primitive and often performed on fixed tissue. The spreading of chromosomes was often inadequate, and they stained very dark; overlapping or juxtaposition made precise counts difficult. Painter was indecisive about the human diploid number but concluded it was between 44 and 48, near to Winiwarter’s finding of 47 or 48. He offered to send fixed testicular tissue to other scientists if they wished to make their own determination of the human diploid number. For another 30 years, the human chromosome number was considered to be 48.^25^,^26^ Painter was, thereafter, connected to the wrong diploid number, an unfortunate stigma for a remarkably productive scientist, elected to the National Academy of Sciences.^24^

In the early 1950s, refinements in cytological techniques were developing, and these stimulated more active interest in cytogenetics. Tao-Chiuh Hsu (1917–2003), born in Chekiang Province in China, immigrated to the United States in 1948 and enrolled in the University of Texas in Austin in a PhD program in which he mapped the chromosomes of the salivary gland in *Drosophila melanogaster*. Painter had become president of the University of Texas by this time, but the program in *Drosophila* genetics at Texas was still pre-eminent. As a post-doctoral fellow, at the University of Texas Medical Branch in Galveston, Hsu changed his interest to those of his supervisor, Charles Marc Pomerat (1905–1964), and studied human cells in tissue culture. Pomerat helped found the Tissue Culture Association and served as its president. Hsu studied chromosome number in human male embryonic skin and spleen cells. Four of four (100%) of skin cell meta-phases and 91 of 124 (73.4%) spleen cell metaphases were found to contain 48 chromosomes. The remaining spleen cell preparations ranged from 44 to 49 chromosomes, and only 11 cells (9%) had 46 chromosomes. Hsu published these results in the *Journal of Heredity* in 1952, perpetuating the concept that the human diploid number was 48.^27^ During these studies, he accidentally discovered the effects of pretreatment of cells with hypotonic fluid on the improved separation and morphology of chromosomes in a spread preparation. Inadvertently, hypotonic Tyrode’s solution was used in place of isotonic saline to wash cells under study before fixation. Hsu noticed the improved spreading and morphology of chromosomes that resulted. The hypotonic wash resulted in spreading of the chromosomes, minimizing their overlapping, such that they could be counted more accurately, their length measured, their long and short arms displayed, and the position of the centromere determined more readily. Despite Hsu’s error in counting chromosomes, which confirmed de Winiwarter’s and Painter’s erroneous conclusions that the human diploid number was probably 48, he went on to a successful career. Among other accomplishments, he froze fibroblasts of mammalian species from the aardvark to the zebra (A to Z), a project featured in *Time* magazine in 1971 as the “Frozen Zoo”, which he did with the prospect that future cloning of endangered or extinct species might become a reality,^28^ a prescient concept at that time. Text-books and review articles in respected journals considered 48 the human diploid number into the mid-1950s, and that number was taught to biology and medical students as late as 1956.

The human diploid number of 48 was first challenged by Joe Hin Tjio (1919–2001) and Johan Albert Levan (1905–1998) studying fibroblasts in four human embryonic lung specimens. In 1956, they published their findings that showed that 252 of 256 human cells contained 46 chromosomes.^29^ Initially there was a question as to whether gonadal tissue studied by Winiwarter and Painter could have a different chromosomal number than somatic tissues. In November 1956, Charles E. Ford (1912–1999) and John Laurence Hamerton (1929–2006) put that concern to rest when they reported that the modal haploid chromosome number in human spermatogonial tissue was 23.^30^ It later was learned that several scientists had studied the chromosome number in human tissue cells and found 46 chromosomes but presumed they had used an erroneous technique; the number 48 was so firmly established.^29^ Indeed, a chromosome spread provided by Hsu used in the book by Cyril Dean Darlington (1903–1981), the Sherardian chair of botany at Oxford University, entitled *The Facts of Life* and published in 1953, had on close inspection 46 chromosomes in the human cell depicted.^29^ In 1957, Hsu and his mentor Pomerat reported a correction to Hsu’s prior work, agreeing that the human diploid number was 46. Tjio and Theodore Thomas Puck (né Puckowitz)(1916–2005)^31^ later published a study showing that the chromosome number in various male and female human tissues was 46. Puck, who received his undergraduate and graduate degrees from the University of Chicago, was a pioneer of cell culture and a renowned geneticist. Until his collaboration with Tjio, Levan had also believed that the human diploid number was 48 based on his own prior studies.^32^ A controversy developed before publication of Tjio and Levan’s report since it was customary in Sweden to list the laboratory director (Levan) as first author, but Tjio insisted that the first author be the principal contributor to the work. In the end that sequence was used and was appropriate since this landmark work is considered to have resulted from the interest and the technical innovations and skill of Tjio.^26^

Tjio was born in Java, Indonesia to Chinese parents and was educated in Dutch colonial schools, which required that he learn French, German, and English, in addition to Dutch. He trained in agronomy in college. He was studying potato breeding, in an effort to develop a hybrid that was resistant to disease. In 1942, Tjio was imprisoned by the Japanese Imperial Army, which had occupied Indonesia. For 3 years, until World War II ended, he was interned in a concentration camp, enduring harsh treatment. When the war ended, a ship sponsored by the International Red Cross resettled displaced persons from Indonesia to The Netherlands. There he obtained a government-sponsored fellowship. He spent several months at the Royal Danish Academy in Copenhagen and then continued his studies and research at the University of Lund in Sweden, where he began an association with Levan, director of the Institute of Genetics. He had also continued his studies on plant and insect cytogenetics and was an authority in the field. The government of Spain invited him to conduct studies on a plant-improvement program, and he spent 11 years in Zaragoza assisting Spanish agricultural development. During holidays, however, he carried out research at the Institute of Genetics, where he turned his attention to mammalian tissue. Tjio became interested in the question of whether consistent chromosomal changes were associated with malignant tumors and returned to Sweden each summer to pursue that work with Levan. Tjio and Levan improved Hsu’s method of pretreating cells with a shortened exposure to hypotonic solution and by adding colchicine to the cell culture medium prior to fixation, thus getting sharper images of chromosomes in metaphase.^29^,^31^

With improved techniques and the human haploid and diploid numbers settled, examination of mammalian chromosomes became an important technique in cell biology, medical research and diagnosis, and cancer cell research. The ability to distinguish deviations in chromosome number and shape, accurately, resulted in a series of important discoveries. Diagnostic abnormalities of chromosome number described in Down’s, Turner’s, and Klinefelter’s syndromes in 1959 propelled the field of medical genetics. In 1960, Peter Carey Nowell (b. 1928) and David Alden Hungerford (1927–1993), working in the pathology department at the University of Pennsylvania, reported the shortening of the long arm of a small chromosome (either number 21 or 22 they initially thought) in seven consecutive patients with chronic myelogenous leukemia. According to the convention of the time, it was named the Philadelphia (Ph) chromosome for the city in which it was discovered.^33^ This finding propelled cancer cytogenetics as a research and diagnostic tool. Today, the cascade of events that followed the discovery of the Philadelphia chromosome has provided a profound advance in cancer diagnosis and the pharmacological treatment of cancer.

## DEFINING THE SIGNIFICANCE OF THE SEX CHROMATIN BODY

Approximately 10 years passed from the observation of Barr and his colleagues in 1949 that a sex chromatin body was present in the nucleus of the cells of females. The question of the nature of the chromatin body was advanced in 1959 when Susumu Ohno (1928–2000) reported his studies of liver cells in *Rattus norvegicus.*^34^ Here he disputed Barr’s idea that the nuclear satellite was the reflection of the two X chromosomes in apposition. He felt this unlikely because heterochromatin is usually only evident on one X chromosome. Moreover, examination of interphase nuclei indicated that the sex chromatin body was smaller than would be the case if it represented two heterochromatic X chromosomes, but was of similar size to one X chromosome.

**Figure 3:**
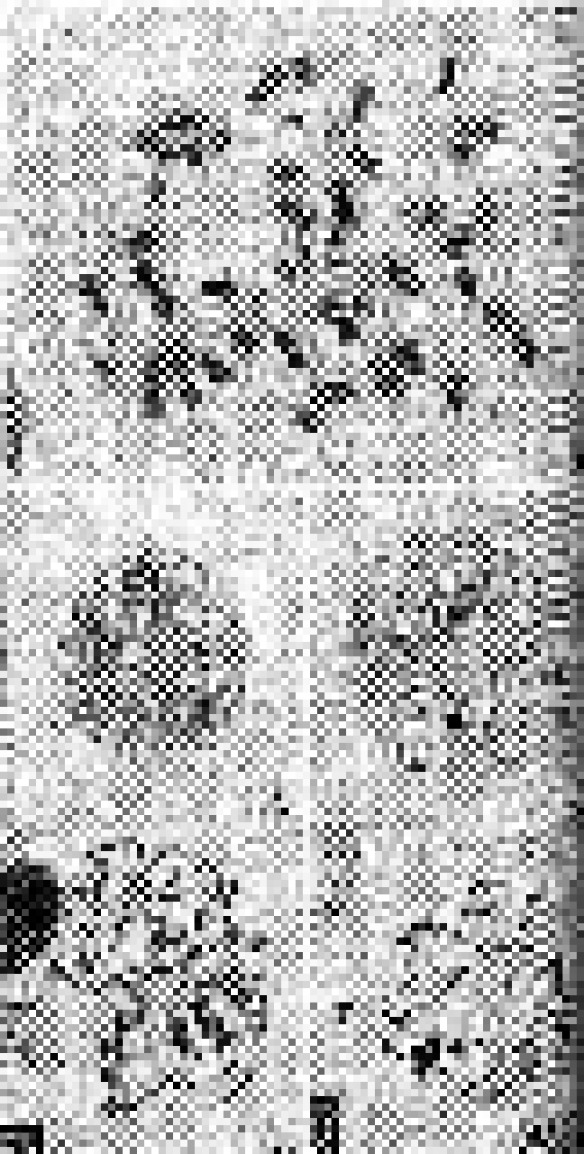
Photomicrographs (× 1,200) of liver parenchymal cells from human fetuses at about fourth month of gestation. A: Female metaphase figure with 46 chromosomes; B: male interphase nucleus with a small chromocenter, presumably representing the Y, discernable at top center; C: female interphase nucleus with sex chromatin body plainly visible in lower center; D: male prophase nucleus with a small chromosome in center distinctly showing positive heteropyknosis (the inset in the lower left corner shows a small acrocentric metaphase chromosome presumed to be the Y); and E: female prophase nucleus with one large chromosome at the lower left demonstrating precocious condensation (the inset in the lower left corner shows the submediocentric metaphase chromosome presumed to be the X). (From: Ohno S, The Lancet 1961;1(7168):78, with permission from Elsevier.^38^)

In 1958, Tjio working with a colleague at the Karolinska Institute in Stockholm concluded that the sex chromatin was the result of viral parasitism of the chromosome by the Bittner mammary tumor agent virus.^35^ This report prompted Ohno to collaborate with Theodore Spaeth Hauschka (1908–1999), using the latter’s mouse models of the milk-agent strains, various other mouse strains infected with other tumor viruses, and uninfected strains. In 1959, they reported that the sex chromatin body in the cells of female mice was the nuclear reflection of a heterochromatic X chromosome during interphase. This finding was confirmed in diploid and heteroploid cells, in non-malignant and malignant cells, and in cells from various tissues, and was independent of infection with the Bittner mammary gland tumor virus.^36^ Ohno initially surmised that the heterochromatic X chromosome was derived from the paternal X because one X in females and the Y in males were paternally derived, but he revised that opinion when he learned that the single, paternally derived X chromosome in the somatic cells of the X^p^O female mouse did not show heteropyknosis.^37^,^38^ He, thereafter, thought that the heterochromatic X chromosome could be either maternally or paternally derived. He and a colleague also studied the hepatic cells of human fetuses while Ohno was on a sabbatical leave in Japan and showed that the sex chromatin body represented the heterochromatic X chromosome in human cells (Figure 3).^38^

By 1959, it was established 1) that one X chromosome in the normal mammalian female’s intermitotic cell is heterochromatic and the other X chromosome is isochromatic with autosomal chromosomes, and 2) that the heterochromatic X chromosome accounts for the nuclear structure referred to as the nuclear sex chromatin body, originally described by Barr and Bertram. Barr met Ohno at a meeting of the American Genetics Society where Ohno presented his findings establishing that the nuclear sex chromatin represented one heterochromatic X chromosome. Barr, after appreciating Ohno’s insights, wrote a letter to the journal *Science* in which he affirmed that Ohno’s hypothesis was correct, remarking,“We are now passing from the descriptive to the more difficult analytical phase in the study of the sex chromatin.”^16^

Ohno was born into an aristocratic Japanese family. His father was the minister of education of the Japanese Viceroyship of Korea. His love of horses led him to veterinary school, graduating from the Tokyo University of Agriculture and Technology in 1949, the year Barr reported his findings. In 1953, he received his PhD degree from the Hokkaido University Faculty of Science. Recognizing the future of science was most promising in the United States, he immigrated and did post-doctoral work at the University of California at Los Angeles. His mentor accepted a position at City of Hope Medical Center in Duarte, California, in the newly organized department of research. Ohno accompanied him and spent the rest of his scientific career at that institution, where he began to study chromosomes and genetics more generally. Ohno was among the most insightful geneticists of his era.^39^ His proposal on the origins of the X and Y chromosomes from ancestral autosomes, his explanation of the role of duplication of genes in the process of evolution, and his explanation of the role of “junk DNA” (a term he coined) in genome evolution were described in his papers and in two monographs, *Sex Chromosomes and Sex-Linked Genes,* published in 1967, and *Evolution by Gene Duplication*, published in 1970. Ohno’s research and his interpretations and insights resulted in his expounding universal principles, a reflection of his remarkable intellect.

Hauschka, a graduate of Princeton University and the University of Pennsylvania graduate school, worked at the Institute for Cancer Research at Fox Chase in Philadelphia and later at Roswell Park Memorial Institute in Buffalo, New York. The Institute was named for Buffalo’s most distinguished surgeon of the early twentieth century and originated from a grant awarded by New York State to establish a pathology laboratory to study cancer. It was the first governmentally funded effort to study those diseases.(Later the Institute’s name was changed to Roswell Park Cancer Institute.) Hauschka, a cancer biologist and geneticist, was studying the chromosomes of cancer cells in mouse ascites tumors. He also was a pioneer in the study of inbred mice and the factors involved in tissue transplantation. In particular, he had developed or established mouse tumor models of various types that were widely used by collaborating scientists at a time when cancer cell lines were uncommon. These mouse models provided Ohno with the opportunity to show, conclusively, the fallacy of the concept that the Bittner virus formed the sex chromatin body.

In 1960, studies in hamster cells by James Herbert Taylor (1916–1998), using tritiated-thymidine labeling and autoradiography of dividing cells, showed that one X chromosome in female cells was late-replicating, both starting and finishing replication later than other chromosomes.^40^ He believed that the late-replicating chromosome was the heterochromatic X. Indeed, a similar pattern had been reported in 1959 in grasshopper chromosomes in which the author proposed that heterochromatin duplicates later than euchromatin. In 1964, studies in human cells by Avery Abba Sandberg (b. 1921) and colleagues, working at the Roswell Park Memorial Institute, found that one X chromosome in women’s cells replicated late in the DNA synthesis phase of the mitotic cycle, and it was the heterochromatic X chromosome during interphase. Sandberg, founding editor of the journal *Cancer Genetics and Cytogenetics*, is a pioneer of cytogenetic studies and cancer cytogenetics and the sole author of the first comprehensive text-book on the subject, *The Chromosomes of Human Cancer and Leukemia*, first published in 1972 and revised in 1980.

With some exceptions, allelic genes on autosomes transcribe and encode proteins. In the case of the X chromosome, if such was the case, females would be encoding twice the amount of gene product as males. Genes in the heterochromatic areas of chromosomes of lower forms did not transcribe, and it was thought possible by Ohno that the genes on the heterochromatic X could be largely silent, resulting in gene dosage regulation in female cells. In that case, only the genes on the active X chromosome would encode proteins, in effect providing both male and female cells with similar dosage effects of most X chromosome-linked genes.

## MOUSE COAT COLOR VARIEGATION AND X CHROMOSOME INACTIVATION

At the time Ohno was working on the anatomical basis of the sex chromatin and opining on its significance, a mouse geneticist, Mary Lyon (b. 1925), was pondering the genetic basis of the aberrant coat colors in mice, variably referred to as mottled, brindled, tortoise-shell, or dappled. Lyon was educated at Girton, a college for women who matriculated at Cambridge University.^41^ At the time she matriculated in 1946, a Cambridge University degree was not available to women, although the curriculum, instruction, and examinations were the same, a situation rectified a few years later. After completing her “titular” degree, she started her post-graduate studies in zoology at Cambridge but moved to the University of Edinburgh, in part because it had better facilities and programs in mouse genetics and there were mentors in place with more similar interests to hers. In 1950, she was awarded the PhD degree and continued post-doctoral work at Edinburgh. In addition to other mouse genetic studies, she began examining the effects of radiation on induction of mutations in mice.

Three events had propelled an interest in radiation mutagenesis in the late 1940s and early 1950s. First, the Nobel Prize in Physiology or Medicine was awarded to Herman Joseph Muller (1890–1967) in 1946 for the first demonstration of radiation-induced mutagenesis, accomplished in *Drosophila* (in 1932 Muller had discussed what he termed “dosage compensation” to explain the equality of phenotypic expression of genes on the X chromosome in males and females); second, the effects of the establishment of the nuclear energy industry with hundreds of thousands of workers exposed to radioactive materials and the detonation of two atomic bombs and the casualties and fatalities that resulted in 1945 in Japan; and third, the cold war between the Western democracies and the Soviet Union and the countries it dominated in Eastern Europe, which resulted in the race by both sides to outdo the other in accumulating nuclear warheads and their delivery systems. There was, not unexpectedly, impetus and grant money to examine the deleterious effects of ionizing radiation and methods to minimize them.

At the same time, interest in genetics as a discipline and the nature of the physical and functional characteristics of the gene was becoming central to biology. The experimental technique of X-ray mutagenesis had led to several monumental advances in understanding gene action. As an example, George Wells Beadle (1903–1989) and Edward Lawrie Tatum (1909–1975) shared the Nobel Prize in Physiology or Medicine in 1958 for a series of illuminating studies of radiation mutagenesis in the bread fungus *Neurospora crassa* in which they discovered the role of genetic regulation of specific enzymes in metabolic pathways in cells. Their work resulted in the dictum “one gene, one enzyme”, which had to be modified later but served as an important operational principle for decades. It also was the experimental confirmation of Garrod’s insights 50 years earlier in his writings about inborn errors of metabolism.

**Figure 4:**
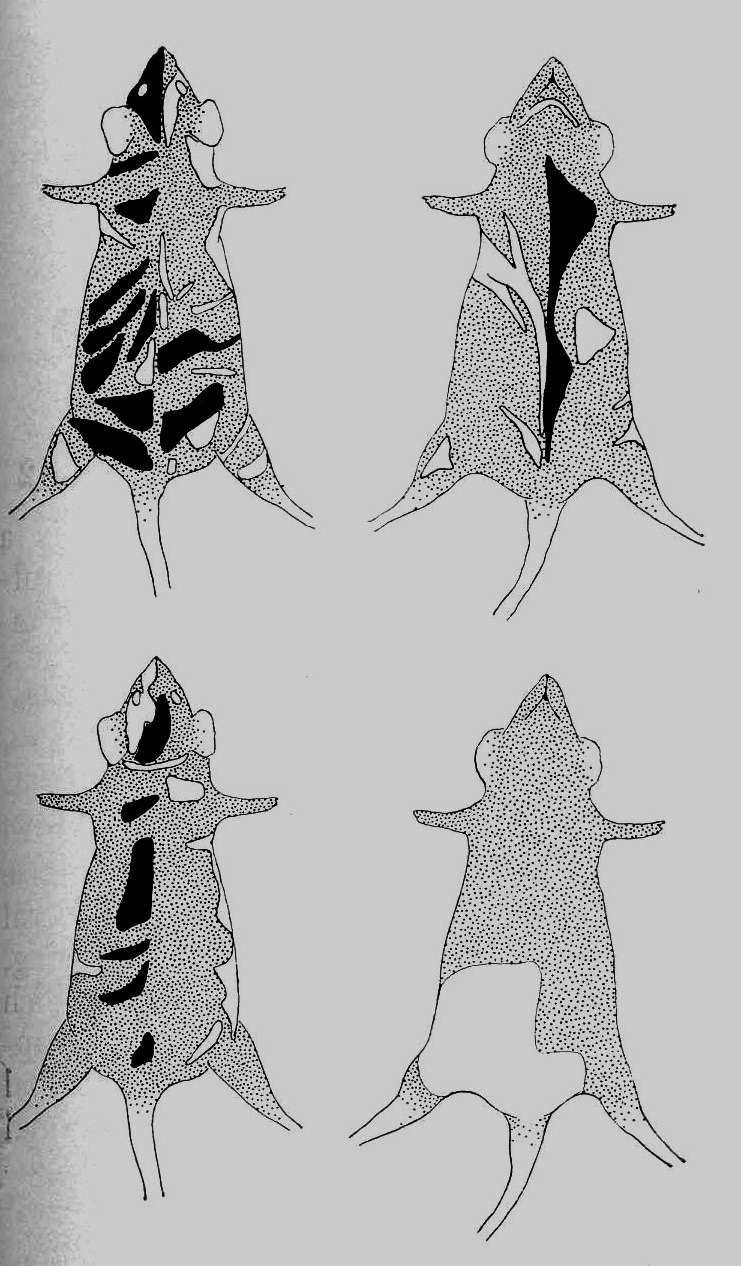
Diagrams show the dorsal and ventral sides of two typical dappled-2 females. The black areas denote full-colored and the stippled areas denote light-colored patches. Note that patches rarely cross the mid-line. (From: Lyon MF. J Hered 1960;51:116–21. with permission from Oxford University Press.^42^)

In the early 1950s, Lyon moved from Edinburgh to the Medical Research Council facility in Harwell, Oxfordshire, England where the national Atomic Energy Research Establishment had been developed to study the (harmful) effects of radiation, especially cancer onset. Lyon’s supervisor and mentor at Edinburgh was Douglas Scott Falconer (1913–2004) whose group had found the first sex-linked coat color mutants in mice, such as mottled (*Mo*). Lyon continued to work with these mutants at Harwell. In one strain, females were mottled and males died early in embryogenesis (a lethal mutation). In an allelic mutant, brindled (*Mo^br^*) males lived long enough (about 2 weeks) to show the wild-type coat color (white); the variegated coat pattern, as in other mouse coat color mutations (e.g. tortoise-shell, dappled), was X chromosome-linked. Falconer gave Lyon a fertile male with a spontaneous mutation (not irradiated) with mottled skin coloration.^42^ Lyon bred from this mouse and established that the skin pattern was inherited and the offspring patterns were consistent with X chromosome-linked coat color transmission among these mice (Figure 4).

The finding of a mottled male was puzzling. Lyon considered that this odd event could develop if a spontaneous mutation occurred early in embryogenesis at the stage of a very few cells. In such a case, some cells would continue to proliferate and differentiate carrying the normal X chromosome and another population of cells would proliferate and differentiate carrying the somatically mutated X chromosome. If this were the case, this male could be a mosaic of a normal and somatically mutated X chromosome, despite inheriting one X chromosome. Lyon considered that if this phenomenon explained the mottled appearance in this unusual male, perhaps an analogous event explained the coat appearance in mottled female mice. In such a female, however, two X chromosomes would be present but X chromosome inactivation early in embryogenesis, as suggested by Ohno’s explanation for the sex chromatin body, would result in clones of skin cells with a normal X chromosome and clones with a mutant X chromosome. The variegated skin color could be the phenotypic expression of some clones of skin cells carrying the wild-type gene on their X chromosome and some clones carrying the mutant coat color gene on their X chromosome.

At about the time Lyon was studying mouse coat color in 1959, William Lawson Russell (1910–2003) and Liane (Lee) Brauch Russell (b. 1923) showed that an XO female mouse could develop normally. Also, the XO mouse did not have the sex chromatin body in their cells.^37^ In 1960, Ohno and Hauschka had shown that the cells of normal female mammals had one hyperchromatic X chromosome.^36^ Lyon considered the evidence that only one X chromosome is required to generate a normal female mouse and that the sex chromatin represented a hyperchromatic and possibly genetically inactive maternal or paternal X chromosome and deduced that, depending on which X was active in a particular clone of skin cells, patches of wild-type (white) color would be interspersed with patches of mutant coloration. In 1961, she articulated her hypothesis in a short article in the journal *Nature*, opining that the hyperchromatic X chromosome was inactive (did not encode protein) and that this inactivation occurred randomly early in embryogenesis resulting in female somatic cells expressing the genes on either the maternal or paternal X chromosome, but not both.^43^ The next year, in a comprehensive discussion of what was known about sex-linked traits in mammals, Lyon extended her hypothesis concerning random X inactivation to all mammals including humans. She stated:

This paper describes in greater detail a hypothesis, which has already been put forward briefly, concerning gene action in the X chromosome of the mouse, and at the same time extends it to cover the X chromosomes of mammals generally. The hypothesis was formed by the welding together of facts recently described in the two fields of mouse genetics and mouse cytology. The hypothesis is put forward that the normal method of dosage compensation in mammals, including man, is inactivation of one of the two X chromosomes of females. It is postulated that the inactive X forms the sex chromatin body, that either one of the two Xs may be inactivated in different cells of the same animal, and that the inactivation occurs early in development…Recent discoveries concerning sex chromosome abnormalities in man suggest that in such cases all X chromosomes in excess of one normal one are inactivated. This hypothesis also explains some hitherto puzzling facts, such as the viability of XXXXY individuals…^44^

The concept of X chromosome inactivation in females became known as the “Lyon hypothesis” in recognition of her proposal and the process of random inactivation referred to by the slang term “lyonization”. The hypothesis is now a theory.

In 1961, Lee Russell summarized, virtually simultaneously (July 1961) with Lyon (April 1961), the progress that had been made on the cytogenetics of mammalian sex chromosomes. In this article, Russell, a mouse geneticist at Oak Ridge Laboratories, Oak Ridge, Tennessee, elaborated on how V-type (variegated type, a term adopted from *Drosophila* genetics) position effects, observed in mice, had contributed to contemporary understanding of the X chromosome, illustrating this effect with female mice carrying a translocation between chromosome 8 and an X chromosome that resulted in a coat color that consisted of discrete brown and wild-type (white) patches. Here she gives Ohno credit for his important intellectual contribution to the concept:

The disproportionately great power of the X to produce such effects in the mouse would seem to indicate that there may be very little heterochromatin on the autosomes, while the X is strongly heterochromatic. The work of Ohno and his collaborators brings cytological corroboration of this conclusion, which we drew on genetic grounds…Certain females have occurred in our stocks which, although they carried the X-8 translocation, were wild-type rather than variegated. Breeding tests revealed that these animals lacked the intact X chromosome – that is, they were essentially XO. Thus, females with the translocation and an intact X (that is, carrying a total of two Xs) are variegated; males with the translocation and the Y (carrying a total of one X) are wild-type; and females with the translocation but lacking the intact X (carrying a total of one X) are also wild-type. We therefore conclude that the presence of two X chromosomes is necessary for the expression of the V-type position effect.^45^

Russell further elaborated on the matter with reference to its implications in humans:

In man, cytological investigations on normal individuals, as well as on patients with an abnormal number of sex chromosomes, have indicated that the number of heteropyknotic chromosomes at somatic prophase, or of “sex-chromatin-bodies” at interphase is, in general, always one less than the number of X chromosomes present. These various findings permit the hypothesis that, in mammals, genic balance requires the action of one X in a manner which precludes realization of its heterochromatic potentialities, so that only additional Xs present assume the properties characteristic of heterochromatin.^45^

Lee Brauch was born in Vienna and immigrated to the United States. She and her future husband, William Russell, who had immigrated to the United States from England, received their PhD degrees in zoology at the University of Chicago. After their marriage, they worked at the Jackson Memorial Laboratory in Maine; they subsequently accepted positions in the Biology Division of the Oak Ridge National Laboratory in 1947. Each, independently and collaboratively, pursued their interest in genetics. In the 1950s Lee Russell and her colleagues established that the mammalian Y chromosome determined the male phenotype and that the karyotypes XO or XX resulted in the female phenotype. Their studies of X-autosome translocations contributed to the evidence resulting in the hypothesis of X chromosome inactivation in females and the explanation for variegated female mice heterozygous for coat color gene mutations. Her contributions to genetics and radiobiology led to many honors, including the Enrico Fermi Prize in 1993. Her contributions to understanding mammalian mutagenesis and applications to genetic risk assessment were extraordinary. Her husband was President of the Genetics Society of America and also won the Enrico Fermi Prize as well as other honors for his contributions to the discipline.

## X CHROMOSOME INACTIVATION IN WOMEN AND DISEASE EXPRESSION

Up to this point, 1961, there was inferential evidence of mosaicism for X chromosome-linked traits in women, but these observations were descriptive and, although highly suggestive in some cases, were inconclusive as reviewed by Lyon in her article on gene inactivation of the mammalian X chromosome.^44^ Ernest Beutler (1928–2008), a physician-scientist and hematologist, working at the City of Hope Medical Center in Duarte, California, was interested in the metabolism and diseases of the red cell. After finishing medical residency at the University of Chicago, Beutler entered the US Army Medical Corps during the Korean War. A need arose to develop better prophylaxis and treatment of malaria to protect American servicemen stationed in Korea. Beutler was assigned to this project. The research was conducted on informative inmates of African descent at the Joliet Prison in Illinois. The problem under study was that a subset of men of African descent given the principal anti-malarial, primaquine, developed an acute hemolytic anemia a day or two after ingestion, making the use of primaquine in a large segment of the troops in Korea problematic. The question being pursued was the pathogenesis of the hemolytic anemia in susceptible individuals exposed to primaquine. It was appreciated at about the same time that ingestion of several drugs and fava beans could also induce acute hemolytic anemia. The Malarial Research Project was begun during World War II and was conducted by the Department of Medicine at the University of Chicago in conjunction with the United States Army. The United States armed forces were exposed to malaria in the African and Pacific theaters during World War II, which was an important factor in impairing combat ability. Tens of thousands of US troops died of malaria in the Mediterranean and the Pacific theaters, and many more were incapacitated. During the Korean War, many troops were infected with malaria. The need for human subjects to test new anti-malarial drugs was met by taking the research into the prison system. At Joliet Prison, over four hundred inmates had volunteered for the project, which involved exposing them to *Anopheles* mosquitoes infected with plasmodia in the laboratory.

In 1956, Alf Sven Alving (1902–1965) and co-workers, at the University of Chicago, proved that the red cells of primaquine-sensitive individuals were deficient in the enzyme G6PD,^46^ and in 1958 Ruth Taubenhaus (Toby) Gross (1920–2007), a pediatrician at Stanford, along with colleagues at Columbia University College of Physicians and Surgeons, confirmed that G6PD deficiency was inherited, as had been previously suspected, and that the mechanism of inheritance was X chromosome-linked.^47^ During the 1950s, Beutler and his colleagues at the University of Chicago completed what became a classic series of experiments defining the nature of the hemolytic anemia in primaquine-sensitive persons. In 1960, Beutler was offered and accepted the chair of the department of medicine at City of Hope Medical Center. By this time, he had become focused on the red cell, hemolytic anemia, iron metabolism and its diseases, and genetic aspects of hematological diseases.

Beutler and Ohno were now both working at the City of Hope Medical Center, but the two did not know each other. Beutler was encouraged to seek out Ohno by his friend and colleague Arno Motulsky (b. 1923) when they were together at the VIII International Congress of Hematology in Tokyo, Japan in September 1960.^48^ Motulsky knew Beutler and was familiar with his work on G6PD deficiency and his interest in the variability of its expression. Motulsky thought that Ohno would be helpful in questions related to the genetics of G6PD deficiency, especially since it was an X chromosome-linked gene. Both Motulsky and Beutler were German-Jewish refugees who left Europe after Adolph Hitler (1889–1945) came to power. Beutler’s family settled in Milwaukee, Wisconsin. His mother and father were phys-icians, his brother (Frederick) became a professor of mathematics at the University of Michigan, and his sister (Ruth) a clinical psychologist. Beutler entered the University of Chicago after 2 years of high school at age 15 years, as part of an innovative and controversial program established by Robert Maynard Hutchins (1899–1977), then President of the University. In 1939, Motulsky, at the age of 15, left Europe with his mother, brother, and sister on the Steamship (S.S.) St Louis from Hamburg bound for Cuba and then the United States. The approximately 1,000 passengers were denied landing privileges when they arrived in Cuba, where his father awaited their arrival, and also were denied entry into the United States. The ship eventually returned to Antwerp, and Motulsky’s family was granted asylum in Brussels. After a year, the family obtained United States visas, but the Germans invaded Belgium and Motulsky was interned in a series of camps in Vichy, France. He was able to get his visa renewed at the American Consulate in Marseilles and he managed to get to Spain and then to Portugal just before his eighteenth birthday and left from Lisbon for the United States. Generalissimo Francisco Franco (1892–1975) had ordered that men aged 18 years or older could no longer leave Spain. He had just made it. In the United States, he met his future wife, Gretel Stern, who had also escaped Nazi Germany via London, then to New York, and finally Chicago. Motulsky had been drafted into the Army out of college in Chicago and was placed in the Army Specialized Training Program. In this program, he entered Yale University for premedical studies and earned the doctor of medicine degree at the University of Illinois. A residency in internal medicine and a fellowship in hematology followed. Thereafter, he continued his Army service at Walter Reed Army Medical Center doing hematology clinical and research work under the tutelage of William Holmes Crosby (1914–2005), a leading clinical and research hematologist of that time. Motulsky’s research at Walter Reed led to his recruitment to the relatively new medical school of the University of Washington in Seattle. Clement Finch (1915–2010), a distinguished physician-scientist, invited him to join the hematology division that Finch led in 1953. Motulsky was interested in medical genetics, an interest kindled by an undergraduate genetics course taught by Donald Frederick Poulson (1910–1989) at Yale University. Poulson, a developmental geneticist, received his PhD at Cal Tech and was the founding chair of Yale’s department of biology. Motulsky began informally giving genetics lectures to medical students using inherited disorders of blood cells as examples. He was encouraged to start a division of medical genetics in the department of medicine by its chair, Robert Hardin Williams (1909–1979), which he did in 1957, the same year that Victor Almon McKusick (1921–2008) began his department of medical genetics at Johns Hopkins University School of Medicine. Before establishing his medical genetics program at the University of Washington, he visited programs in Europe and the United States, which gave him important insights into the faculty interests needed to have a comprehensive program. Motulsky became the father of the science of pharmacogenetics, a discipline he pioneered.^49^ He received the American College of Medical Genetics Foundation Lifetime Achievement Award in 2009, among several honors for his distinguished career.

Motulsky and Beutler, although at different institutions, were friends and colleagues since both were interested in genetics and hematology and had similar life experiences. In those days, medical academic specialty societies were small and intimate, and most members knew each other. Motulsky’s suggestion that Beutler seek out Ohno had a profound effect on Beutler’s research, and Ohno and his wife Midori and Beutler and his wife Bonnie became lifelong friends. Beutler later credited Ohno with being the “father of [the idea of] X chromosome inactivation”.

Virgil Fox Fairbanks (b. 1930), who is presently Emeritus Professor of Medicine, Laboratory Medicine and Pathology at the Mayo School of Medicine and Consultant at the Mayo Foundation Clinic, first encountered Beutler in 1960 at a meeting of the Western Society for Clinical Research in Carmel, California. Fairbanks was then in the midst of a hematology fellowship at the Scripps Clinic in La Jolla, California. He had been working on red cell enzymology and glycolytic metabolites. Beutler was in great need of staff on his arrival at City of Hope and he found Fairbanks an attractive recruit as their interests meshed. Fairbanks spent three-and-a-half years working with Beutler as a clinical research associate. Fairbanks recalls the circumstances that led to their landmark 1962 publication “The normal human female as a mosaic of X-chromosome activity: studies using the gene for G6PD deficiency as a marker”,^50^ the authorship of which included their laboratory technologist Mary Yeh.

**Figure 5:**
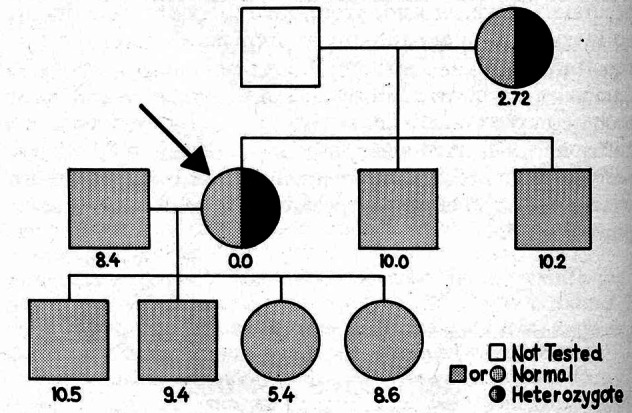
Pedigree of index case (arrow). Figures beneath each member of kindred re-present activities of G6PDH. Note the index case has no measurable G6PD, although she has two sons with normal G6PD activities. Older daughter of index case has erythrocyte G6PD level which is at lower limit of normal. She may also be heterozygote, although this cannot be established at present. (From: Trujillo J, The Lancet 1961;ii:1445, with permission from Elsevier.^52^)

Fairbanks related that a patient on the medical service at University of California at Los Angeles had a perplexing, recurrent severe acute hemolytic anemia, requiring red cell transfusions. William Newton Valentine (b. 1917), a hematologist expert in hemolytic anemias, chief of hematology, and later chair of medicine, was asked to see the patient. The patient was a Sephardic Jewish woman who lived in North Hollywood. Valentine quickly identified the basis of the problem: she ate fava beans. She was diagnosed with fava-bean-induced acute hemolytic anemia, known by then to be related to a red cell deficiency in G6PD. Beutler and Valentine were friends and colleagues. Valentine contacted Beutler to suggest he study this patient and her family. Beutler was interested and asked his associate, Fairbanks, to collect blood samples from the index case and the members of her family. Fairbanks, subsequently, obtained blood from 20 family members and spent so much time with the family that he learned about Sephardic Jewish culture and about the origins of Ladino, which was the language they spoke to each other.^51^ Fairbanks did all the assays on the family on June 30th, 1961. (He retained and re-examined his laboratory note-books at our request.)In a letter to the journal *Lancet* in 1961, Beutler, Fairbanks, and colleagues described the family pedigree and ascertained that despite the unexpected complete absence of G6PD activity in the red cells of the propositus, it was not possible for her to be homozygous for G6PD deficiency because she had two sons with normal red cell G6PD levels (Figure 5).Fairbanks recalled that each time he measured her red cell G6PD level the spectrophotometer registered slightly below zero. She also had a sex chromatin body in her buccal mucosal cells and a normal karyotype.^52^ According to Fairbanks, Beutler and he puzzled as to how the propositus, a woman and heterozygote, could be totally deficient in red cell G6PD. It was a frequent topic of conversation between them until as Fairbanks recalls “the light came on one day”, and he (Beutler) said: “Aha, I’ve got it…it is X chromosome inactivation, but instead of a 50:50 inactivation the X chromosome with the normal allele for G6PD has been inactivated in all her red cell precursors, leaving the expression of only the mutant gene!” Beutler reasoned that occasionally the inactivation would be in a skewed ratio, in this case approximating 100:0, resulting in exaggerated expression of the mutant X chromosome and profound susceptibility to hemolysis.^51^ Beutler credited his colleague at City of Hope, Ohno, with having prepared his mind to understand X chromosome inactivation as the explanation for the variable expression of X chromosome-linked traits in women. Fairbanks recalled that Ohno’s discussion of the heterochromatic X had an important influence on the thought processes of Beutler, and presumably Lyon, since Ohno’s paper is the first cited in the publication describing her hypothesis in the journal *Nature.*^43^

Beutler and Fairbanks had to establish that women were mosaics for X chromosome-linked genes. Whereas mice have an X chromosome-linked gene that determines pigmentation (coat coloration) that can be used in studies, as was done by Lyon, human epidermal cells migrate differently during embryogenesis than do pigment cells, making mosaicism very difficult to assess. Beutler considered red cell G6PD “an ideal test system” through which to evaluate the hypothesis that human females represent a genetic mosaic. Published in the journal the *Proceedings of the National Academy of Sciences* in January 1962, the paper presented a series of experiments designed to determine the kinetics of red cell G6PD enzyme activity in women heterozygous for the gene.^50^ Consistent with their hypothesis, the authors were able to demonstrate that women heterozygous for G6PD deficiency have two distinct erythrocyte populations, one with normal enzyme activity (active X chromosome without mutation expressing G6PD in an average of half of the erythroid progenitor population) and the other with absent enzyme activity (active X chromosome carrying mutation failing to express G6PD in half of the erythroid population).

**Figure 6:**
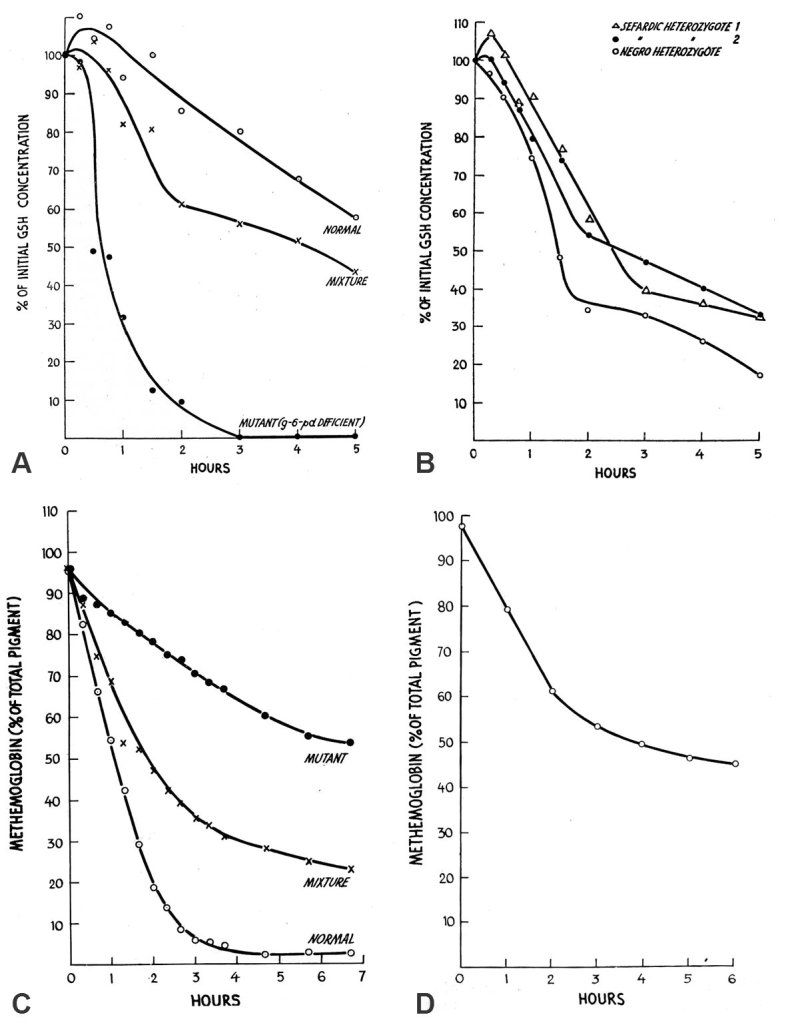
A: The rate of disappearance of glutathion(GSH) from the blood of a G6PD-deficient male, a normal subject, and an artificial mixture. One-cc samples of whole blood were incubated with 5 mg acetylphenylhydrazine. Each point represents the average of duplicate determinations. B: The rate of disappearance of GSH from the blood of three females with “intermediate” blood G6PD levels. C: The rate of methemoglobin reduction in the red cells of a G6PD-deficient male, a normal subject, and an artificial mixture. The reaction mixture, incubated at 37°C, contained the following: glucose 5 mg/cc; Nile blue sulfate 11 μg/mL; red blood cells 29% suspension; all in isotonic potassium phosphate buffer at pH7.4.D: The rate of methemoglobin reduction in the red cell of a Negro female with “intermediate” red cell G6PD activity.(From: Beutler E, Proc Natl Acad Sci U S A 1962;48:9, with permission from the National Academy of Sciences.^50^)

G6PD catalyzes the first enzymatic step inthe pentose phosphate pathway, transforming glucose-6-phosphate to 6-phosphogluconic acid, generating reduced nicotinamide adenine dinucleotide phosphate (NADPH), which must be generated in order to maintain a supply of reduced glutathione in the red cell. Beutler and colleagues showed that when incubated with the oxidizing agent acetylphenylhydrazine the rate of decline in glutathione in the blood of females heterozygous for the G6PD gene approximated that of an artificial equal mixture of type-compatible blood from a normal subject and from a G6PD-deficient male (Figure 6). Both samples tested generated a two-component curve, with a sharp inflection in red cell glutathione between the first and second components. This result, as opposed to the generation of “a single curve of intermediate slope” from assaying the blood of G6PD heterozygous females that would be expected if these subjects had a single population of erythrocytes with intermediate enzyme activity, strongly supported the concept of two distinct red cell populations in these subjects, one with an active and one with an inactive X chromosome in their erythroid precursor cells.

Beutler’s laboratory provided further evidence in support of this concept by measuring the rate of reduction of methemoglobin in nitrite-treated red cells in the presence of Nile blue sulfate. Nile blue sulfate reduces methemoglobin; its ability to do so is linked to the availability of the end-products of the pentose-phosphate shunt and is therefore dependent on G6PD activity. As predicted, a two-component curve represented the rate of reduction of methemoglobin in a sample that consisted of an artificial mixture of erythrocytes from a G6PD-deficient male with those from a normal subject as well as in a sample from a female heterozygous for G6PD deficiency (Figure 6).

**Figure 7:**
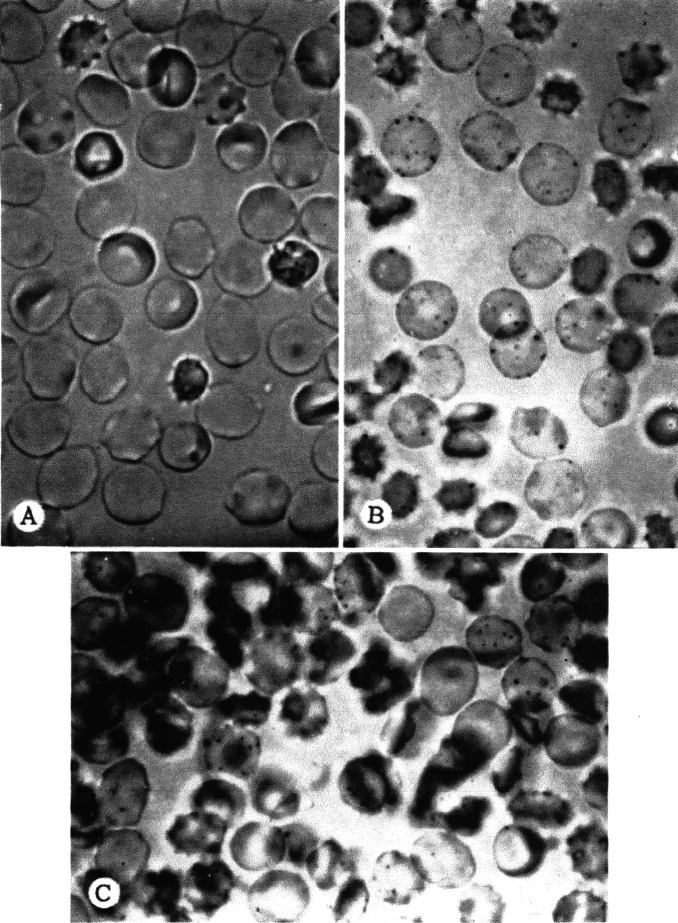
Intact erythrocytes. A: Negro male with marked G6PD deficiency. All erythrocytes are devoid of formazan granules. B: Caucasian male with spectrophotometrically normal G6PD activity. All erythrocytes contain formazan granules. C: Chinese female with intermediate expression of G6PD deficiency. Mosaicism is easily seen in that many cells have abundant formazan granules, and many are devoid of granules (×1,000). (From: Fairbanks VF. Blood 1968;31(5):589-603, with permission from the American Society of Hematology.^54^)

Fairbanks and Beutler designed a quick, cheap, and simple method of detecting G6PD deficiency based on the reduction of a tetrazolium dye, which turns purple in the reduced state.^53^ Later, Fairbanks and a colleague described a method of staining individual erythrocytes for G6PD, based on the reduction of a tetrazolium salt to a chromogenic formazan dye. G6PD activity could be graded based on the number of purple granules produced, allowing direct visualization of the G6PD level in individual erythrocytes (Figure 7). Fairbanks stated: “Mosaicism is readily recognized by this technique; many heterozygotes with spectrophotometrically normal levels of G-6-PD have thus been identified. The application of this technique therefore provides additional strong evidence, by a relatively direct approach, of X chromosome mosaicism in the human female.”^54^

For several years there was a commercially available kit for G6PD deficiency screening using the method developed by Fairbanks and Beutler. Subsequently, Beutler developed a screening test based on the fluorescence of NADPH in ultraviolet light. NADP does not fluoresce. G6PD reduces NADP to NADPH, which exhibits strong fluorescence in ultraviolet light.

In a presentation given at the IX Congress of the International Society of Hematology in September 1964 in Mexico City, Beutler summarized his observations (made jointly with Fairbanks) concerning G6PD as a marker of X chromosome mosaicism. He discussed the contributions of Ohno to their work, crediting him with making the astute observation that one of the X chromosomes of mammalian females is hyperchromatic, producing the sex chromatin in the interphase nucleus of female cells in mammals.^55^ The latter concept that an X chromosome-linked gene in any particular mammal would be so in others became known as Ohno’s Law. As part of this concept, Ohno predicted that the establishment of dosage compensation contributed to stabilization of the gene content of the X chromosome. In the presentation, Beutler stated that “these findings suggested independently to us and to Mary Lyon in England that perhaps only one X-chromosome could be active in directing protein synthesis.” They elaborated upon their hypothesis that the human female is a genetic mosaic in that she has two populations of cells, one of which has an active maternal X chromosome and another with an active paternal X chromosome. The statement they made was in agreement with the hypothesis proposed by Lyon:

We should like to propose that when the egg is fertilized by an X-chromosome-bearing sperm, both X-chromosomes remain active for a number of divisions. At the morula stage of embryonic development, the Barr body appears. We would suggest that at this stage of development the non-homologous portion of the X-chromosome is inactivated, at least partially. We would propose that inactivation of X-chromosomes takes place at random, the maternally derived X-chromosome being inactivated in some cells, the paternally derived X-chromosomes being inactivated in other cells. From this stage on only one X-chromosome is active in directing protein synthesis in each cell and this is the only active X-chromosome in the entire clone of cells produced from this precursor cell. Thus, the normal human female may be considered to be a mosaic of clones of cells, some containing an active maternally derived X-chromosome, others containing an active paternally derived X-chromosome… The hypothesis that Dr Lyon and we have proposed appears to us to explain most satisfactorily the lack of dosage effect and the marked variation and expression of heterozygotes for glucose-6-phosphate dehydrogenase deficiency and other sex-linked traits. While it must be admitted that the evidence that we have obtained that there are two red cell populations in heterozygotes for G-6-PD deficiency is indirect, we have found no experimental situation in which the cells of heterozygote G-6-PD deficiency behave differently than an artificial mixture of mutant and normal erythrocytes.^56^

In 1962, two phenotypic variants of red cell and leukocyte G6PD in American men and women of African descent not deficient in G6PD were described on the basis of the rate of their electrophoretic migration. Compound heterozygotes for the two electrophoretic variants were found in American women of African descent but not in American men of African descent, providing further evidence that the gene for G6PD is X-linked.^57^ In 1963, investigators were successful in finding laboratory evidence supporting random X inactivation in females using gel electrophoresis. The skin cells of females heterozygous for G6PD deficiency were shown to have two G6PD bands on electrophoresis, whereas cells cloned from a culture derived from a single skin cell had only one of these two bands. These investigators commented: “The appearance of two distinct populations of cells in the female heterozygous for both quantitative and qualitative G6PD variants is direct evidence in favor of the ‘Lyon Hypothesis’. As far as the locus for G6PD is concerned, in each single cell only one X chromosome is functional.”^58^

Lyon published her hypothesis based on her insights and studies in mice in the April 22, 1961 issue of the journal *Nature*. Beutler and Fairbank’s work was transmitted to the *Proceedings of the National Academy of Sciences* on November 30, 1961 by Sturtevant and was published in January 1962. Forty years earlier, Sturtevant had been a student in Morgan’s “Fly Lab” in which location the first sex-linked trait in *Drosophila* was discovered. Indeed, the history of discovery and exploration of X chromosome inactivation is replete with close professional interactions or those of only a few degrees of separation.

## BIOCHEMICAL VERIFICATION OF X CHROMOSOME INACTIVATION

In the late 1970s, a series of papers, using biochemical techniques, found that both X chromosomes remained active in mouse embryos until the mid-cleavage stage, but by day 6.5 post-coitus, at the onset of gastrulation, one X chromosome was inactive in somatic cells.^59^ In the mid-1990s, a transgenic mouse model, using the *E. Coli LacZ* gene linked to a mouse housekeeping gene generated a β-galactosidase cell marker. Studies in this mouse showed a somewhat different schedule of X chromosome inactivation in different tissues. Somatic cells in these mouse embryos had two active X chromosomes during the period 5.5–8.5 days post-coitus. The two active X chromosomes did not interfere with tissue development at that stage. X chromosome inactivation was required, however, for normal differentiation and development of mesodermal and ectodermal tissue.^59^ A mouse model of X chromosome-autosome translocation had shown severe abnormalities in ectodermal and mesodermal development if two X chromosomes remain active.

X chromosome inactivation requires a remarkable sequence of events. The somatic cell must recognize the presence of two X chromosomes and choose one to inactivate in a manner that results in the paternal or maternal X chromo-some being inactivated with a probability of approximately 0.5. An epigenetic or genetic mechanism or both must propagate X chromosome gene silencing, and silencing must be maintained in the descendants of the cell. The X inactivation center (Xic) silences adjacent DNA sequences. Although cis-acting elements are dominant in X chromosome inactivation, trans-acting factors have been described, and epigenetic factors (e.g. DNA methylation) are critically important in silencing.^60^

## X CHROMOSOME INACTIVATION AS A MARKER FOR THE CLONAL ORIGIN OF TUMORS

Fairbanks recalls accompanying Stanley Michael Gartler (b. 1923), a geneticist at the University of Washington, to obtain skin biopsies from the same Sephardic family on which he and Beutler had studied the inheritance of the gene for G6PD deficiency.^51^ Gartler earned a PhD in plant genetics from the University of California at Berkley and obtained post-doctoral training at the Institute for the Study of Human Variation at Columbia University. While at Columbia, Gartler met Motulsky who was in New York recruiting persons to join his new genetics unit in the department of medicine at the University of Washington. Motulsky was interested in recruiting scientists and physicians with various areas of expertness in the newly developing areas of cytogenetics and biochemical genetics, among others. Gartler accepted the opportunity to become more involved in studies of human biochemical genetics. Gartler explained that he became interested in G6PD deficiency not because of an interest in X chromosome inactivation per se but rather as a potential marker for clonality in studies of tissue cell development.^61^ In the case of the Sephardic family being studied by Fairbanks and Beutler, Gartler was using skin biopsies to develop a technique to clone skin fibroblasts; he hoped to validate the presence of a clone by assaying the level of G6PD activity.

David Linder (1923–1999), who was a pathologist at Children’s Hospital in San Francisco with very heavy clinical responsibilities, applied for and received a 1-year National Institute of Health fellowship that was designed for physicians to have an opportunity to conduct research and to encourage research careers. Linder decided to take his fellowship in the Department of Pathology at the University of Washington. He discussed his interests with various faculty members within and outside of the department. He found Gartler’s ideas regarding studies of the cellular origin of tissues intriguing. Linder had the idea of using G6PD isoenzymes to explore the question of the single cell origin (somatic mutation hypothesis) of neoplasms as contrasted with the field theory of carcinogenesis. He decided to use leiomyomas for these experiments and to compare them to intervening normal myometrium in informative women. Gartler remarked that there was no human investigation committee at that time, and no permission was required to use human tissue that was to be discarded. Linder, as a pathologist, simply arranged to obtain excised uteri from the surgical pathology laboratory for their studies of patients of African descent who had undergone a hysterectomy for “fibroids”, not an uncommon event.^61^ They first homogenized a portion of the myometrium and did gel electrophoresis to determine if the individual in question was informative, i.e. a compound heterozygote for G6PD isoenzymes A and B.

**Figure 8:**
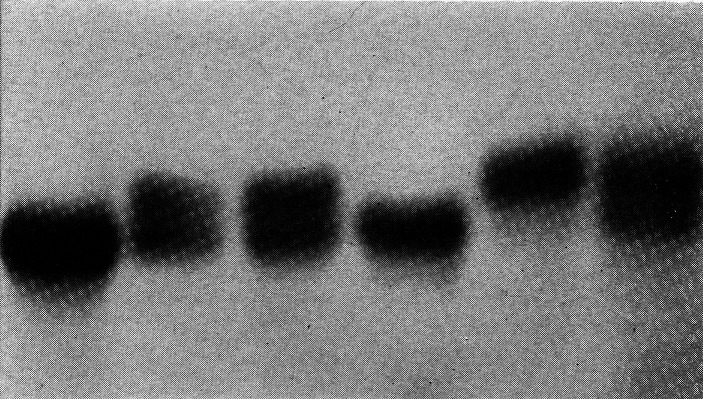
Electrophoresis of tissue samples from normal myometrium and leiomyomas in one individual. Slots from left to right: 1st, B tumor; 2nd and 3rd, AB normal myometrium samples; 4th, B tumor; 5th, A tumor; 6th, AB normal myometrium sample. (From: Linder D, Science 1965;150:67-9, with permission from the American Association for the Advancement of Science.^62^)

In 1965, Linder and Gartler reported their ingenious studies of the G6PD isoenzymes in uterine tissue from five women of African descent who were compound heterozygotes at the G6PD locus. These five women had cells containing G6PD type A encoded by the gene on one X chromosome and G6PD type B encoded by the gene on the alternative X chromosome. They measured G6PD electrophoretic phenotypes in tissue from leiomyomas and normal intervening myometrial tissue. Whereas the samples of normal uterine tissue exhibited both G6PD isoenzymes A and B (distinguished by the appearance of two bands migrating through a starch gel electrophoresis), the samples from leiomyomas had either G6PD isoenzyme type A or B, but not both (Figure 8). Although there were several interpretations of this finding, the authors felt the most likely interpretation of their data was in support of the hypothesis that leiomyomas and, by extension, other neoplasms were derived from somatic mutations in a single cell.^62^

After completion of his year in Gartler’s laboratory, Linder returned to his position as a clinical pathologist in San Francisco, later moving to the department of pathology at the University of Oregon. While in San Francisco he and Gartler extended their studies, greatly increasing the number of leiomyomas and myometrial samples analyzed and did other analyses that permitted them to conclude to a degree of scientific certainty that uterine myomas originated in a single cell.^63^ Linder, who had suffered from poliomyelitis as a child, died at a relatively young age as a consequence in part of the post-polio syndrome.^62^ Gartler described Linder as curious, bright, and tenacious and encouraged him to stay in research, but his family’s desire to return to San Francisco held sway. The findings and methods established, however, led to further studies of the cell of origin of neoplasms in Gartler’s laboratory.

After completing his internship and first residency year at the University of California at San Francisco, Philip Jack Fialkow (1934–1996) continued his residency training at the University of Washington in Seattle. Developing an interest in human genetics, he pursued a 2-year fellowship in Motulsky’s division of medical genetics.^61^ Fialkow worked in the Gartler laboratory, applying Linder’s and Gartler’s technique of using G6PD isoenzyme expression to study the cell of origin of chronic myelogenous leukemia. Akira Yoshida (1924–2005), a protein chemist and geneticist who had been recruited from Japan by Motulsky, was asked by Gartler to collaborate in the project that Fialkow was undertaking. In 1972, Yoshida moved to the City of Hope Medical Center and served as its director of the Department of Biochemical Genetics from 1981 to 1994.

Fialkow, Gartler, and Yoshida published their findings in 1967. They isolated fibroblasts, granulocytes, and erythrocytes from females of African descent with chronic myelogenous leukemia who were heterozygous for G6PD isoenzymes A and B, separating the isoenzymes by gel electrophoresis. Whereas fibroblasts were found to express both G6PD isoenzymes, granulocytes and erythrocytes were shown to express the same single isoenzyme type. These results had 2-fold significance. They were consistent with a monoclonal origin of chronic myelogenous leukemia and with the cell of origin being a common multipotential hematopoietic cell capable of differentiating into either an erythrocyte or a granulocyte. The authors considered alternative explanations of their findings to be less probable, concluding:“it appears most likely that this malignancy arises as a consequence of a rare event in a single stem cell.”^64^ This finding was compatible with the presence of the Ph chromosome in all lineages of a multipotential hematopoietic progenitor (or possibly stem) cell. In a relatively meteoric rise, Fialkow, who joined the faculty of the University of Washington medical school in 1965, rose to the rank of full professor in 8 years, went on to become head of the department of medicine at the University of Washington, and then dean of the medical school. Tragically, Fialkow and his wife died in an avalanche while hiking in Nepal in the fall of 1996.^65^

## NEW TECHNIQUES FOR THE IDENTIFICATION OF X CHROMOSOME INACTIVATION

G6PD activity was informative in a small proportion of women who were heterozygous for isoenzymes A and B. As more was learned about X chromosome function and genetics, new methods, informative in most women, were developed. Several very difficult assays were developed in the 1970s; one required one X chromosome to be morphologically distinguishable from the other. Another method required the preparation of rodent-human hybrid cells. The latter was technically complicated, expensive, and took weeks for the read-out. The first practical method was devised by Bert Vogelstein (b. 1949), a molecular oncologist at Johns Hopkins Medical Center, who used a cloned polymorphic X chromosome gene, hypoxanthine phosphoribosyltransferase (*HPRT*), and two restriction endonucleases for the assay. The first endonuclease distinguished the maternal and paternal copies of the gene through a restriction fragment length polymorphism. The second endonuclease distinguished the active from inactive copy of the gene, through its methylation status. If a tumor developed from one cell – that is, if it were monoclonal –the paternal copy of the gene would be cleaved by the second enzyme in a much different fashion from the maternal copy, since the paternal copy was either active in all the cells of the tumor or inactive in all the cells of the tumor. In normal tissue, approximately half the cells had an active paternal gene and half had an active maternal gene, so that the paternal and maternal copies of the gene were affected similarly by digestion with the second enzyme.^66^ This assay was succeeded by the use of the human androgen receptor as the informative gene.^67^ An adaptation using the androgen receptor gene was developed in which a methylation-specific polymerase chain reaction served as the basis for the assay for the clonal derivation of tissue cells.^68^ A technique based on X chromosome-transcribed allelic ratio has been proposed as a more specific approach to detecting clonality in the tissues of women, a method less susceptible to false skewing.^69^

With the passing of time the knowledge regarding the mechanisms of X chromosome inactivation accumulated. The role of the epigen-ome in silencing had been described.^70^,^71^ In each succeeding decade after Lyon published her conceptual paper and Beutler published his studies of mosaicism of X chromosome-linked G6PD in the human female, extensive reviews of the advances in understanding of the genetic and molecular aspects of inactivation were published. One decade after her seminal report in 1961, Lyon reviewed advances in the study of X chromosome inactivation.^72^ Approximately a decade later, in 1983, Gartler reviewed subsequent advances.^73^ In the early 1990s, Arthur D. Riggs and Gerd P. Pfeifer at City of Hope Medical Center,^74^ Gartler,^75^ and Lyon^76^,^77^ reviewed the continued progress in the study of X chromosome inactivation.

## SEQUENCING THE X CHROMOSOME AND ITS SPECIAL FEATURES

In 2005, Mark T. Ross and 282 colleagues at 21 collaborating institutions determined the DNA sequence of the human X chromosome, identifying 1,098 genes covering at least 99.3% of the length of its euchromatin.^78^,^79^ That number of genes reflects a gene-poor chromosome containing numerous interspersed repeat sequences. In 1998, Lyon hypothesized that repetitive long interspersed nuclear elements (LINEs) of the L1 family exist along the X chromosome, which, when transcribed, are responsible for facilitating X chromosome inactivation.^80^ Ross found a high distribution of L1 sequences in close proximity to the X chromosome inactivation center (Xic), the region on the X chromosome from which inactivation originates. The inactivation center is at band q13 and a gene, X-inactive specific transcript (*XIST*), sits at the inactivation center. *XIST*, a non-coding RNA gene, is present on the inactive X chromosome and is important in the execution of inactivation.^81^ The non-coding RNA genes are not included in the total number of genes assigned to the X chromosome because of the difficulty distinguishing genes from pseudogenes.^78^

The inactivation process is complex. Some proportion of genes (∼15%) on the inactive X chromosome continue to encode proteins,^82^–^85^ and it has been proposed that individuals with abnormalities resulting from non-disjunction, who have a sex chromosome complement that includes extra X chromosomes, may have phenotypic manifestations from the expression of multiple non-silenced allelic X chromosome-linked genes.

Because inactivation occurs at a stage in which only a small number of cells are involved, a random event can result in a markedly skewed pattern of inactivation by chance. Thus, the phenotype of an X chromosome-linked gene mutation may be similar in the female to that of the affected hemizygous male (as observed by Beutler and which led to his further, now landmark, studies, see Figures 5 and 6), or, conversely, the phenotype of a heterozygous female may be virtually normal. This effect is the result of the binomial probability of inactivation. Thus, the average expectation of a 50:50 distribution of maternal and paternal active X chromosomes in the tissue of a female is just that, an average. The distribution may be 60:40/40:60, 70:30/30:70, and so on, including 100:0/0:100.

The inactive X chromosome is reactivated during meiosis in the oocyte and undergoes recombination with the second X chromosome so that the zygote receives two active X chromosomes, and the process of inactivation is repeated, thereafter, during embryogenesis. The Y chromosome does not recombine with the male X chromosome along most of its length but does so at their tips, areas referred to as “pseudoautosomal”.

## Abbreviations:

G6PD: glucose-6-phosphate dehydrogenase;
NADPH: nicotinamide adenine dinucleotide phosphate;
SRY: sex-determining region Y gene;
Xic: X inactivation center;
XIST: X-inactive specific transcript;
